# Why do rural women in the most remote and poorest areas of Zambia predominantly attend only one antenatal care visit with a skilled provider? A qualitative inquiry

**DOI:** 10.1186/s12913-018-3212-9

**Published:** 2018-06-05

**Authors:** Choolwe Jacobs, Charles Michelo, Mosa Moshabela

**Affiliations:** 1 School of Nursing and Public Health, University of KwaZulu-Natal, Durban, South Africa; 2 School of Public Health, Department of Epidemiology & Biostatistics, Section for Surveillance & Disease Control, University of Zambia, Lusaka, Zambia; 3 Strategic Centre for Health Systems Metrics and Evaluations (SCHEME), School of Public Health, University of Zambia, Lusaka, Zambia; 4 Africa Health Research Institute, Durban, Kwa-Zulu Natal South Africa

**Keywords:** Antenatal care, Appreciative inquiry, Access to care, Healthcare utilisation, Rural, Remote, Zambia

## Abstract

**Background:**

While focused antenatal care (ANC) has served as an entry point in the continuum of care for both mothers and children, fewer than a third of pregnant women in the most remote and poorest communities of Zambia achieve the four ANC visits recommended by the World Health Organization. Current evidence suggests that attending ANC provided by a skilled healthcare worker at least once is common and associated with skilled birth attendance. The aim of this study was to explain why one ANC visit with a skilled provider seemed more common than four ANC visits among women in the remote and poorest districts of Zambia.

**Methods:**

A qualitative case study design was conducted in 2012 among 84 participants in the selected remote and poorest districts of Zambia. Focus group discussions were conducted with mothers and community health volunteers, while key informant interviews were conducted with healthcare providers. Thematic analysis was conducted.

**Results:**

Most women delayed starting antenatal care visits due to uncertainties about the timing for initiation of ANC and due to waiting for confirmation of the pregnancy by an elderly woman. Attendance of ANC once with a skilled provider was due to the need to assess their health status and that of their baby. In some facilities, attendance of ANC at least once was enforced by financial charges imposed on women for late ANC initiation, and/or incentives provided by nongovernmental organisations. Unavailability of services at health posts closest to these remote communities led to failure to return for subsequent ANC visits. Women’s livelihoods such as nomadic lifestyles made it harder for them to initiate and make additional ANC visits.

**Conclusion:**

The popularity of ANC attendance once by a skilled provider among the remote and poorest women of Zambia was explained through perceived unavoidable social and economic barriers to care, and the punitive and incentive procedures implemented by health services. Maximising comprehensive care by skilled healthcare workers in the one visit a woman makes at the health facility, may lead to optimal utilisation of quality focused ANC. Enhancing community-based interventions may increase the potential to reach the vulnerable populations.

**Electronic supplementary material:**

The online version of this article (10.1186/s12913-018-3212-9) contains supplementary material, which is available to authorized users.

## Background

The World Health Organization (WHO) recommends focused antenatal care (ANC) as an essential approach to the care of pregnant women [1]. The main goal of the WHO’s focused ANC is to facilitate early detection and treatment of pregnancy-related complications with the minimum number of visits [2]. Each visit includes care that is appropriate to the woman’s overall condition and stage of pregnancy and facilitates preparation for birth and care of the newborn. According to the WHO [2], antenatal care provides a platform for important healthcare functions, including health promotion, screening and diagnosis, and disease prevention.

When utilised, focused ANC can improve women’s likelihood of accessing skilled birth attendance [3] and postnatal care [4], and improve maternal and newborn health outcomes [5, 6]. For instance, some studies have shown that receiving focused ANC from a skilled health attendant is associated with better infant care practices, increased use of postnatal check-ups and decreased risk of both maternal and neonatal mortality [7–9]. Conversely, women who attend fewer than four ANC visits were less likely to receive skilled attendance at delivery [10, 11]. In Egypt, a study among high-risk women (pre-eclamptic) who did not attend the WHO-recommended four ANC visits had a 12-times greater risk of poor maternal outcome, a 53-times greater risk of poor fetal outcome, and a significantly high risk of neonatal mortality, compared to women who attended the recommended four antenatal visits [11].

Zambia, like many developing countries, adopted the WHO model of focused ANC in 2003 which entails that the expectant woman visits the health facility a minimum of four times, at 16 weeks, between 24 and 28 weeks, at 32 weeks and the last one at 36 weeks, for women whose pregnancies were progressing normally [12]. Focused ANC also requires that for each visit a woman makes she should be attended by a skilled healthcare provider [13]. While focused ANC has served as an entry point in the continuum of care for both mothers and children [14], the achievement of ANC at least four times is not common in many low- and middle-income countries, including Zambia [12–15]. It was demonstrated in a recent survey that less than a third of pregnant women in the most remote and poorest communities of Zambia achieved the WHO-recommended four ANC visits, 53% lower than the national estimates [15]. Most pregnant women in resource-poor settings are unable to meet the recommended four ANC visits, largely because of the distances to the health facilities and the poor road conditions [3], delayed initiation of ANC, lower education and income levels [16–19]. These barriers to focused ANC expose women to increased risk of maternal deaths and other pregnancy-related complications [11].

Since 2016, the WHO released new guidelines for focused ANC, recommending a minimum of eight visits during pregnancy from four ANC visits [1]. Zambia is currently transitioning from four ANC visits to eight ANC visits for focused ANC. In a country where four visits are difficult to meet, eight visits will likely be even harder to achieve for these pregnant women. There was, however, a promising observation from our recent survey of the most remote and poorest women in Zambia, in that attendance of ANC with one visit provided by a skilled healthcare worker was more common at 69%, and a significant predictor for skilled birth attendance and postnatal care [15]. There was no explanation found in our survey as to why a single visit with a skilled provider seemed common and positively associated with outcomes among women in the remote and poorest areas. We therefore embarked on an in-depth qualitative study to explain why one ANC visit with a skilled provider seemed more common than four ANC visits among women in the remote and poorest districts of Zambia. Such understanding may help policymakers and implementers in designing appropriate ANC services with a limited number of visits in remote and poverty-stricken communities, without compromising desired outcomes of skilled birth attendance and postnatal care.

## Methods

### Study setting

The study was conducted in four of the most rural and poorest districts of Zambia: Chiengi and Samfya districts from Luapula province, and Mungwi and Luwingu from the northern provinces of Zambia. The selection of these districts was purposive representing the districts classified as poorest, inhabited by the most marginalised people in the country, and with the highest maternal and child mortality rates [20]. Most people are peasant farmers, engaged in crop and livestock production as well as fishing. Due to fishing as the main livelihood for many households, most women and their families are usually away for extended periods of time and only return home occasionally. Most of the surface areas in these districts have poor terrain and are covered by lakes, rivers and hills. For instance, in the Samfya district, approximately 40% of the surface area is covered by wetlands. The total estimated population size of these four districts is 580,090 inhabitants. In Zambia, the health system is organised into different levels of health facilities for service provision. Maternal health services are provided at health post level (the lowest level), health centre level and at the level one (district), two and three (tertiary) hospitals. In the selected sites, women had a choice to attend ANC from either the health post or health centre closest to them.

### Study design

The present research uses a qualitative case study design conducted between August and October 2014.

### Participants and recruitment

Triangulation of different participant groups was used for cross verification and complementary purposes. Women of reproductive age, community healthcare workers, traditional birth attendants, neighbourhood health committee members, and healthcare providers were identified and recruited for participating in the study. They were from one health facility in each of the districts that showed the worst outcomes of maternal health indicators. In each of the four districts, a medical officer, a clinical care expert and a maternal and child health coordinator were purposively selected as key informants at district health office level. Within each of the selected health centres, a health centre-in-charge was purposively sampled for in-depth interviews. Women of reproductive age, with children under one year old and living within the study community during their most recent pregnancy, were included for focus group discussions (FGDs) from each health facility. Community health volunteers that have been actively working for more than six months within the communities under study were purposively sampled and included for FGDs with the help of healthcare workers at the facility level. A total of 84 participants were interviewed within 24 interviews, which included eight FGDs with women (4) and community health volunteers (4). Sixteen key informant interviews (KIIs) (4 from each district) were also conducted. None of the participants approached refused to participate. Table 1 summarises the list of interviews and instruments that were used to collect information.

**Table 1:** List of the respondents who participated in the qualitative study

Type of Tool	Respondents	Number of Interviews (Key Informat Interviews)	Number of participants per interview	Total number of participants
Key Informat Interviews	Medical Officers, Clinical Care Experts and Maternal and Child Health Cordinator	12 (3 in each of the 4 districts)	1	12
	Health Facility in-charge	4 (1 in each of the 4 districts)	1	4
Focus Group Discussions	Mothers of children below one year	4 (1 in each of the health facilities from each district)	8–10	38
	Community Health Volunteers (CHWs, NHC, TBAs)^a^	4 (1 in each of the health facilities from each district)	6–8	30
Total			24	84

^a^*CHW* Community Health Workers, *NHC* Neighbourhood Health Committee, *TBAs* Traditional Birth Attendants

### Data collection

Focus group discussions and KIIs were conducted in English and/or in a local language, Bemba, by trained research assistants experienced in qualitative research. Each FGD was conducted by a pair of research assistants, who were of the same gender and were conversant with the local language. One research assistant facilitated the sessions, while the other one managed the audio recordings and took field notes. The research assistants underwent a one-day training prior to data collection and were supervised by one of the co-authors (CJ). The data collection tools were piloted in a similar facility not included in the study. The average duration of FGDs and KIIs was 45 min. The interviews were delivered at the health facilities on a face-to-face basis, and were intended to gather information on the perceptions of the respondents regarding ANC visits by women, including the frequency of care and reasons why a single visit from a skilled provider seem more common among women than four ANC visits. For this study, a skilled healthcare provider was defined as an accredited health professional, such as a midwife, doctor or nurse who has been “trained to proficiency in the skills needed to manage normal (uncomplicated) pregnancies, childbirth and the immediate postnatal period, and in the identification, management and referral of complications in women and newborns” [21]. An Interview Guide that directed FGDs and KIIs is shown in the Additional file 1. All interviews were digitally recorded after obtaining informed consent from the participants. No repeat interviews were conducted.

### Data management and analysis

The recordings were transcribed and translated from Bemba into English. Six of the 24 transcripts, randomly selected, were verified by back translation into Bemba for accuracy. Transcripts that were not back translated were also reviewed by listening to the original voice recordings to ensure that they retained the original meanings after translation. The transcripts were not returned to the participants for review because of logistical constraints. The transcribed documents in Microsoft Word were then exported into NVIVO 10 software (QSR International, Melbourne, Australia) for coding. The authors (CJ and MM) generated the codes/themes. An inductive thematic analysis was used through an iterative process, whereby themes were continuously generated, revised, and re-examined to discover and make explicit why women predominantly attend only one ANC visit during pregnancy. Triangulation of the different sources, FGs, KIIs, and field notes were employed to validate the data by using cross-referencing.

## Results

### Characteristics of the participants

The age range of the participating mothers varied from 18 to 45 years. Nearly all the mothers were married (36 out of 38) and unemployed (35 out of 38). About two-thirds of the mothers (27 out of 40) had either not attended school before or completed primary school education. Parity for mothers varied between two and 12 children. Only nine out of the 28 community health volunteers interviewed were male. All healthcare workers interviewed had worked in the study sites for over one year preceding the interviews; 11 out of 16 were female. The age range for healthcare providers was between 28 and 49 years.

In this study, we aimed at understanding why attendance of one ANC visit with a skilled provider seemed more common than the recommended four ANC visits. The following themes as shown in Table 2, emerged from the data: factors that affect the timing of the first ANC appointment, factors that affect failure to return for subsequent appointments, factors that affect both timing and return for subsequent appointments, and opportunities for the intervention to modify the different factors.

**Table 2:** Reasons why one antenatal care visit with a skilled provider seemed more common than four ANC visits among women in remote and poorest districts of Zambia

Theme	Sub-Themes		
	Mothers	Community Health volunteers	Healthcare Provider
1. Factors that affect timing of the first ANC appointment	• Uncertainty in the timing of ANC initiation • Lack of transport	• Waiting for elderly women to confirm pregnancy	• Waiting for elderly women to confirm pregnancy
2. Factors that affect failure to return for subsequent appointments	• Unavailability/Low quality services at health posts closest to women º Inadequate supplies at the health facilities º Inadequate health personnel • Lack of awareness and education about the services	• Low quality services at health posts	• Unavailable and Poor-quality services in the health posts • Inadequate supplies • Unavailable/Inadequate skilled health care providers
3. Factors that affect both timing of the first ANC appointment and return for subsequent appointments	• Livelihoods as a priority Nomadic lifestylesBusy schedules with house chores house chores • Long distances to the health facilities	• Denied subsequent care for ANC • Nomadic lifestyles • Long distances to the health facilities	• Nomadic lifestyles • Seasonal migration to places where they might be no health centres • Lack of transport • Lack of money for transport
4. Opportunities for the intervention to modify factors affecting timing of the first ANC appointment and failure to return for subsequent appointments.	• *Assessment of Health Status* • To confirm if really pregnant • Check the health of the unborn baby • Need to get tested for HIV • Need to receive medication such as IPTp • Protection for the unborn baby • To obtain ANC cards • Financial charges imposed on women for late booking	• *Assessment of Health Status* º To check the health of the mother and their unborn baby • To obtain ANC cards • Incentives provided by NGO	• *Assessment of Health Status* º To confirm if really pregnant • Access for services such as mosquito nets, folic acid and test for HIBV • Incentives provided by NGO

### Factors that affect timing of the first ANC appointment

#### Delayed initiation of ANC

A single ANC visit by a skilled provider was more common than four ANC visits for women in the remote areas due to a limited number of visits made overall. Nearly all the respondents reported that most women sought ANC from the health facility at least once.

Attendance of ANC by most women was characterised by delayed entry for ANC. Most healthcare workers and community health volunteers explained that initiation for ANC visits was delayed because most women were quite silent about their pregnancies until it was visible. Most pregnant women agreed that their ANC booking was delayed due to a shared reluctance to prematurely disclose a pregnancy. One of the community health volunteers had this to say:

*Most of these women will be quiet about their pregnancy, and they wait until it [pregnancy] shows* (FGD, Mungwi, female, NHC).

#### Uncertainty in the timing of ANC initiation

Women’s uncertainty in the timing of ANC initiation, largely indicative of a tension between the health provider messages and individual preferences, also explained why timing for the first ANC appointment was delayed. A 26-year-old woman from Chiengi said:

*Some go at four months, others at six and even at seven, but it is not very clear the exact month but I think it depends on how you feel in the body* (FGD, Mungwi, mother, 20-34 years).

#### Waiting for confirmation of pregnancy by elderly women

The fear of ‘bad luck’ held by women also led to women’s preference to wait until the pregnancy was visible before talking to others, including healthcare providers, about their pregnancy. Most of the women waited for elderly women they trusted to confirm the pregnancy. Both healthcare providers and community health volunteers held similar perspectives and indicated that most pregnant women waited for elderly women to confirm a pregnancy before ANC booking, with the consequence of late initiation into ANC visits. One of the healthcare providers had this to say:

*Most of the women in this place start going for ANC very late, most of them after the second trimester. This is because most of them traditionally believe that they will only come for antenatal care after an elderly woman such as an auntie or grandmother confirms that they are pregnant* (In-depth interview [IDI], Chiengi, midwife, 35-49 years).

### Factors that affect failure to return for subsequent appointments

#### Long distances to the facility

Women had an option of attending ANC at a health post nearer to them, or a health centre further away. In most communities, women were reported to visit the health posts for ANC to avoid traveling long distances to the clinics.

*... most women go to the health post, but very few come to the health facilities because many of them cannot walk to the health facility* (IDI, Luwingu, healthcare worker, 35-49 years).

The long distance to the health facilities influenced whether women could return for subsequent ANC appointments. There was a general perception among all the categories of respondents that the long distances rendered attendance of all four recommended visits for ANC difficult for women, as stated below by a mother:

*What I can say is that the distance from here to the hospital [clinic] is very far, the four times they tell us to come for antenatal care is not easy, again walking back there, especially when you are almost due, the clinic is very far, for me most times I fail to keep coming back because of distance* (FGD, Samfya, mother, 35-49 years).

#### (un) availability/poor quality of services

Most women failed to return for subsequent appointments because the services provided at the health posts were of low quality because of inadequate services provided to women, often provided by inadequately trained personnel. On the whole, most health posts were not managed by skilled health facility staff.

*For some of us, the clinic is far from where we come from and to be honest what you are saying does not happen for some of us ... Those tests are just for others who live close to the clinic* (FGD, Mungwi, mother, 35-49 years).

Further, the quality of health services was also reported to be poor at the health posts due to inadequate supplies. A midwife from Chiengi had this to say:

*The antenatal care we provide at the health post is also questionable because you know, we usually lack certain things, you know the focused ANC we provide should be really focused antenatal, but you find that sometimes you do not have everything* (IDI, Chiengi, midwife, 35-49 years).

As a result, women were compelled to go to the health centres for ANC only once.

### Factors that affect both delayed timing of the first ANC and return for subsequent appointments

#### Livelihoods as a priority

Women’s livelihoods among these remote communities were also another challenge for attendance of adequate ANC visits. Most women and their families were reported to be mobile due to fishing, the major source of their livelihood. This came out more particularly in Samfya and Mungwi where it was reported that many women only came once for ANC and did not have regular ANC visits due to their nomadic lifestyles

*… a large population is mobile due to seasonal migration of people, when you consider the fishing period where men and women and their families move to the fishing camp, and also once they discover that ‘my pregnancy is fine and I’ve been checked and okay, all is fine’, women think they are fine, they do not see any need to come back for ANC, and they disappear* (IDI, Samfya, healthcare worker, 35- 49 years).

### Busy schedules with house chores

The discussions with the women also revealed that the busy schedules with house chores could barely allow women to return to the health facility for subsequent ANC visits, given the distances and the time it took to visit the clinic. Women could not afford not to keep up with their daily house chores.

*Sometimes we get very busy at home such that leaving all the work for the clinic becomes a problem. To be honest for my situation at home, without someone to take care of the children at home and others, it becomes hard to abandon the work* (FGD, Chiengi, mother, 35-49 years).

### Opportunities for the intervention to modify the different factors

#### Need to confirm pregnancy

As soon as the pregnancy showed, the women sought care from a skilled healthcare provider to confirm that they were indeed carrying a baby. The need for most women to confirm pregnancy with a skilled provider was perceived as an important driver of ANC bookings. Once the women were convinced that they were indeed pregnant, most of them did not return for the subsequent visits. This health behaviour was reported by most women in nearly all group discussions.

*We go for ANC because we want to know what is in the abdomen ..., because sometimes you may think you are carrying a baby when in fact you are not* (FGD, Mungwi, mother, 20-34 years).

#### Assessment of health status

The women viewed ANC attendance as an opportunity to confirm if they were really pregnant and to check their own health status and that of the baby. Where health problems are identified, women would welcome the necessary health care to ensure their own health and the well-being of their unborn baby.

*Like for me [number 7] why I come here I would want the nurse to tell me and just to know how the baby is and to know my health because if you are just at home you can’t know whether the baby is healthy or not* (FGD Mungwi, woman 20-34 years).

Furthermore, we also found that most women attended ANC mainly to test for HIV and to know their status. Women believed that once they came to the facility, were tested and found they are HIV positive, they would receive medication and their unborn baby would be protected.

*Even me that’s what I know, that a woman who is pregnant should go to the clinic, so that they test your blood [and] if they find that you have the disease [HIV] they will tell you and put you on medication so that you protect the baby from having the disease* (FGD, Samfya, mother, 20-34 years).

This behaviour was also reported by healthcare workers as well as a community health volunteer.

#### Promotion of antenatal care through punishment/incentives

In some facilities, attendance of ANC was enforced through financial charges imposed on women for late bookings. This was reported by most women and the community health volunteers. For instance, a 20-year old woman reported that her ANC booking after three months of pregnancy invoked a fee charged by their clinic, which necessitated her to book early for ANC to avoid the charge.

*Yes, like my friend has said, we are charged for delaying to start ANC ... they say if you come after three months you should pay 15 kwacha* (FGD, Chiengi, mother, 20-34 years).

We also found that in some facilities, some women sought ANC from a skilled provider, even if it was only once, for fear of being denied subsequent care such as skilled attendance at delivery, if they did not obtain an ANC card.

*We fear not being attended to well or being chased away when we come back because if you have no ANC card, they know for sure that one missed ANC. So, we have to come even if it is once so that at least you get a card* (FGD, Samfya, Mother, 35-49 years).

A community healthcare worker had this to say:

*They know that if they do not attend ANC, they will not get the ANC card, and without the card it becomes a problem at the clinic for delivery ... that is why they make sure they come even if it is once* (FGD, Luwingu, CHW, 35-49 years).

Furthermore, incentives provided during antenatal clinics through skilled personnel from the nongovernmental organisation partners, which included provision of mother and baby packs, baby nappies, soap and mosquito nets, motivated women to attend ANC.

*We have seen the numbers of women attending ANC increase when we distribute incentives, although once, especially with the coming in of partners like MSF, they give out Chitenge materials and baby packs, and that acts also like a motivator to the women* (IDI, Luwingu, healthcare worker, 35-49 years).

## Discussion

This study sought to explain why one visit with a skilled provider was more common than four visits among women in the remote and poorest populations in the selected remote and poorest districts of Zambia. Attendance of ANC provided by skilled personnel, often once, by mothers was to confirm pregnancy and check their own health and that of their unborn child. Attendance of ANC by most women was characterised by delayed entry for ANC booking, uncertainty in the timing of ANC booking and due to waiting for confirmation of the pregnancy by elderly women. In this study, it seems that most women in remote areas in Zambia found it inconvenient to revisit ANC once pregnancy, fetal health and their well-being were confirmed. The study has also revealed that, although facilities in these communities, such as health posts, were available close to the women’s homes, inadequate staff and low quality of services in these facilities hindered women from attending adequate ANC visits from skilled personnel. Women’s livelihoods, such as nomadic lifestyles and household chores, led to their preference for a single visit with a skilled provider. Furthermore, and consistent with other studies [22, 23], due to punitive measures imposed on women by healthcare providers and/or incentives provided by nongovernmental organisation partners, some women attended ANC only once and did not return for subsequent visits.

The observations pertaining to women’s inclination to attend a single ANC visit with a skilled provider are perhaps the most important contribution of this study. Consistent with other qualitative studies in the rural parts of countries in sub-Saharan Africa, it was not surprising that most women from the rural and remote communities of Zambia have minimum ANC visits with skilled personnel due to delays in entry to ANC [24–26]. The persisting uncertainty and confusion about timing for ANC commencement expressed by most women have also been echoed in other similar studies and have been explained as being due to limited awareness and information about the importance of ANC [27], a possible indication of health system failures.

On the one hand, women recognise the need to confirm pregnancy with skilled health providers at the clinic, including their own well-being as well as that of their unborn children. However, healthcare providers in health centres further away from the women’s homes are using punitive techniques to coerce women into ANC enrolment and attendance, as well as using occasional incentives to induce women into ANC. Furthermore, services at health posts closest to the women’s homes are inadequate [28, 29], and often provided by inadequately trained personnel, culminating in the by-pass phenomenon [30, 31], a behaviour in which women travel farther than necessary to obtain health care [32]. A combination of these factors reported by both women and community health volunteers suggests the need to appreciate complex mechanisms involved in ANC utilisation, and to co-design strategies together with the women that would improve continuity of care for maternal and neonatal health.

Specific to these rural and remote communities is the value attached to the livelihoods and instrumental activities of daily living, as well as their nomadic lifestyles, which make both initiation and return visits inconvenient, considering the long travel distances involved. According to Killewo and colleagues [33], daily chores tend to distract women’s health-seeking behaviours through delay in seeking help, particularly among poorer women. Also, research in developing countries has shown that livelihoods may supersede women’s motivation to visit ANC clinics [34–37]. Socially sensitive and appropriate healthcare services, considerate of livelihoods and sociocultural practices, with adequate and effective patient-centred communication strategies by healthcare providers [34], could also help modify health-seeking behaviours.

Consistent with the findings reported in prior studies [38, 39], the perceived need for services by women found in this study, including the need for HIV testing and the need to confirm the health of the mother and baby, provide an important opportunity for health promotion strategies such as the provision of information, education and communication for creating awareness of the importance of other pregnancy-related services. Women’s ability to attend ANC, even if it was once in marginalised and hard-to-reach areas, suggests the need for the provision of comprehensive services as a ‘one-stop-shop’ where as many services as possible are provided during the one visit, as subsequent visits for these women may not be guaranteed [40]. Although this argument may seem to contradict the WHO’s ideal focused ANC visits, now to become eight ANC visits [1], the findings generated from this study demonstrated that for these women, their environment (remoteness) was complex and it dictated their ability to use maternal and neonatal health services.

Our findings must be interpreted in light of some limitations. First, the interviews were conducted at the health centres. Therefore, the opinions of the women could have been influenced by some power relations at the health centres. Second, the study population was extremely rural and poor, and may be limited to similar contexts due to differential contextual contrasts that may have existed. Third, recall bias was a possibility because participants were asked to describe situations as far back as one year.

However, although we thought that these limitations could have been present, we do not think they were important in explaining the findings. First, the finding and explanation are based on different perspectives that are also in agreement, through triangulation of different sources, healthcare workers, community health volunteers and the women themselves. Second, this paper brings out the ‘voices’ of the most marginalised women who are not only poor and living in remote and limited resourced areas but are also from less researched areas. An understanding of the poorest and hard-to-reach communities is fundamental in achieving universal access to care and should not be ignored if universal access to focused ANC is to be achieved. Lastly, this study provides timely insight to the most developing countries, particularly Zambia, which is transitioning from the WHO policy of ‘ideal four ANC visits’, now to become ‘ideal eight ANC visits’, on some factors that limit the numbers of ANC visits among the remote and poorest populations of Zambia. Targeted interventions aimed at reaching the poor and the marginalised remote women need to be co-designed by policymakers and programme implementers, including women themselves, if the new WHO ‘ideal eight ANC visits’ is to be achieved.

## Conclusion

In these remote and poorest populations of Zambia, a minimum of one ANC visit to a skilled provider seemed more popular and feasible for these women due to multiple unavoidable contextual factors, creating an environment of social exclusion that pushes the populations further away from the health system. The findings in this study reflect the interplay of structural constraints and social exclusion of the poorest and remote populations [41]. These findings are suggestive of the need to repackage maternal and neonatal health services based on the available opportunities. In the meantime, it is vital that the single ANC visits women spent with skilled healthcare workers are maximised as they may very likely be the only one a rural woman may attend. The findings of this study also call for a rethink in the stated WHO policies of ‘ideal four ANC visits’, now to become ‘ideal eight ANC visits’, without consideration of the women’s financial, geographical and social factors that dictate levels of access and participation in health care. In addition to current community-based strategies, local women groups who work with policymakers could be used to remodel healthcare services to create the best possible fit between healthcare services and users in remote, rural and poor communities.

## Additional file

Additional file 1: Interview Guide used to direct Focus Group Discussions and Key Informat Interviews. (DOCX 27 kb)

## Abbreviations

ANC: Antenatal care
FGD: Focus group discussion
IDI: In-depth interview
KII: Key informant interviews
WHO: World Health Organization
