# Supplementary material for: Why do rural women in the most remote and poorest areas of Zambia predominantly attend only one antenatal care visit with a skilled provider? A qualitative inquiry
Source: BMC Health Serv Res. 2018 Jun 5;18:409. doi: 10.1186/s12913-018-3212-9 (PMC5989442; doi:10.1186/s12913-018-3212-9)
Supplement: Supplementary file 1 — Interview Guide used to direct Focus Group Discussions and Key Informat Interviews. (DOCX 27 kb) [file 12913_2018_3212_MOESM1_ESM.docx]

**Utilisation of Maternal and Neonatal Health care services among the remote and poorest populations of Zambia**

**Interview guide for mothers with children under one year/Health Care Providers/Community Health Volunteers**

**Antenatal, Delivery and Postnatal Health care**

1. Where do women seek health care when pregnant?
2. When do women start going for antenatal care? How many times
3. How frequent (number of antenatal care visits) do women seek antenatal care during pregnancy? Probe why the stated frequency seem common.
4. From whom do pregnant women seek antenatal care from? **Probe:** for type of care provider
5. What motivates pregnant women to seeking antenatal care for the stated number of visits and from the stated provider? Probe: Number of antenatal care and type of provider
6. What are some of the barriers to seeking antenatal care? Probe: At least four times, more than once?
7. How far is the health facility from your home? ( Probe; Time of travel/Far/Near) (Probe for solutions to challenges)
8. What kind of test do pregnant women get when you are pregnant?
9. What do you know about HIV testing during pregnancy?
10. Where do most women give birth from? Give reasons?
11. Who usually conducts the delivery of babies? (**Probe** TBAs) Probe for reasons
12. Who in the family sleep under a mosquito net? Probe; Do pregnant women sleep under a mosquito net? Why/why not?
13. How often do pregnant women sleep under mosquito net? ( **Probe;** all the time, sometimes) Why?
14. What kind of health care do the mothers and children receive after birth?
15. Where do they get the health care service? **Probe** TBA, Health facility. Probe for reasons
16. What could be the reasons for mothers and children not accessing postnatal checkups? Why?
17. What do you think should be done to improve antenatal care, delivery and postnatal care services?

**THANK YOU FOR PARTICIPATING**
